# Hypofractionated Stereotactic Radiosurgery in a Large Bilateral Thalamic and Basal Ganglia Arteriovenous Malformation

**DOI:** 10.1155/2013/631028

**Published:** 2013-11-06

**Authors:** Janet Lee, Tomoko Tanaka, Steven Westgate, Ashish Nanda, Marshall Cress, N. Scott Litofsky

**Affiliations:** ^1^Division of Neurological Surgery, University of Missouri-Columbia School of Medicine, One Hospital Drive, MC 321, Columbia, MO 65212, USA; ^2^Division of Radiation Oncology, University of Missouri-Columbia School of Medicine, Columbia, MO 65212, USA; ^3^Department of Neurology, University of Missouri-Columbia School of Medicine, Columbia, MO 65212, USA

## Abstract

*Purpose*. Arteriovenous malformations (AVMs) in the basal ganglia and thalamus have a more aggressive natural history with a higher morbidity and mortality than AVMs in other locations. Optimal treatment—complete obliteration without new neurological deficits—is often challenging. We present a patient with a large bilateral basal ganglia and thalamic AVM successfully treated with hypofractionated stereotactic radiosurgery (HFSRS) with intensity modulated radiotherapy (IMRT). *Methods*. The patient was treated with hypofractionated stereotactic radiosurgery to 30 Gy at margin in 5 fractions of 9 static fields with a minimultileaf collimator and intensity modulated radiotherapy. *Results*. At 10 months following treatment, digital subtraction angiography showed complete obliteration of the AVM. *Conclusions*. Large bilateral thalamic and basal ganglia AVMs can be successfully treated with complete obliteration by HFSRS with IMRT with relatively limited toxicity. Appropriate caution is recommended.

## 1. Introduction

Arteriovenous malformations (AVMs) in the basal ganglia and thalamus comprise a small percentage of all AVMs [1–4]. These deep AVMs have a more aggressive natural history with a higher morbidity and mortality than AVMs in other locations [5]. Bilateral basal ganglia and thalamic AVMs are extremely rare. The majority of patients harboring these lesions present with hemorrhage and serious neurologic sequela. Optimal treatment—complete obliteration without new neurological deficits—is often challenging. Stereotactic radiosurgery (SRS) is generally accepted as the first option in treatment [3, 5, 6].

In this report, we present a patient with a large bilateral basal ganglia and thalamic AVM successfully treated with hypofractionated stereotactic radiosurgery (HFSRS) with intensity modulated radiotherapy (IMRT). There have been no other case reports of bilateral AVMs of this size successfully treated with radiosurgery. In particular, the complete obliteration of the AVMs after HFSRS with one year of treatment is noteworthy. 

## 2. Case Report

### 2.1. History and Examination

A 12-year-old male presented to his primary care physician with a 3-month history of progressive gait abnormality. On physical examination, he had a subtle left facial droop and hyperreflexia in the left lower extremity. His gait was mildly ataxic with circumduction of the left leg. He was unable to heel walk on the left, and he had sidestepping with tandem gait. Toe walking was preserved. His motor and sensory exam was otherwise unremarkable. 

**Figure 1:**
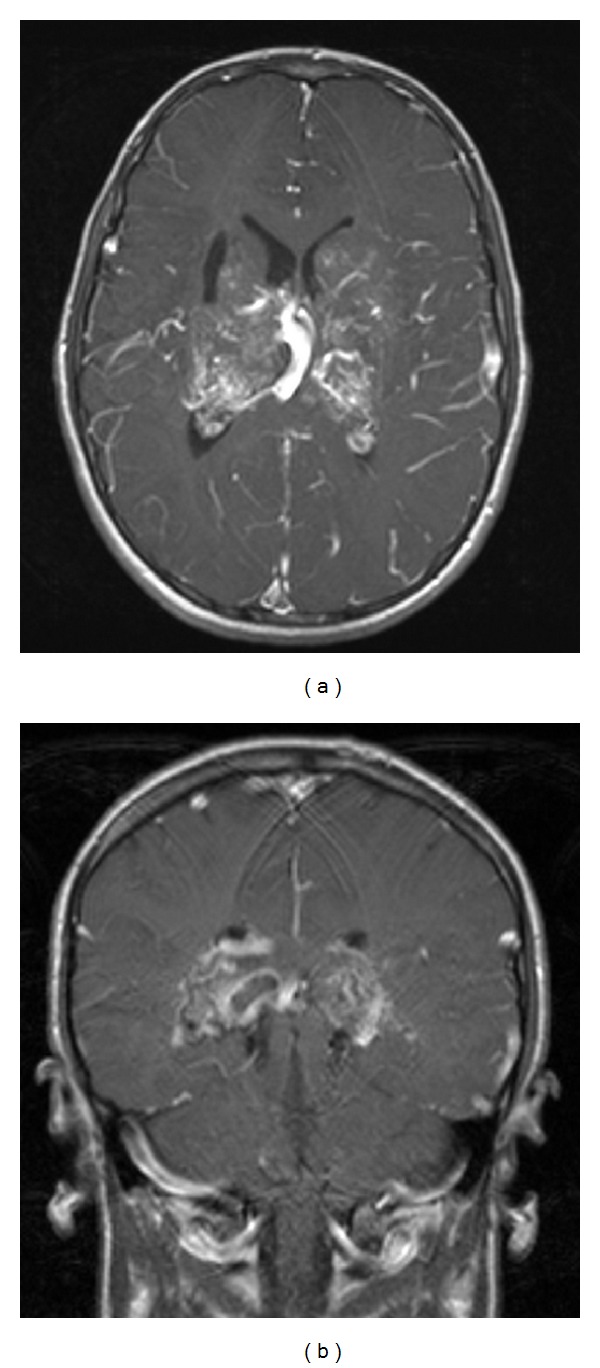
MRI at initial presentation. (a) T1-weighted, axial, gadolinium-enhanced image at the level of the foramen of Monro. (b) T1-weighted, coronal, gadolinium-enhanced image posterior to the third ventricle.

**Figure 2:**
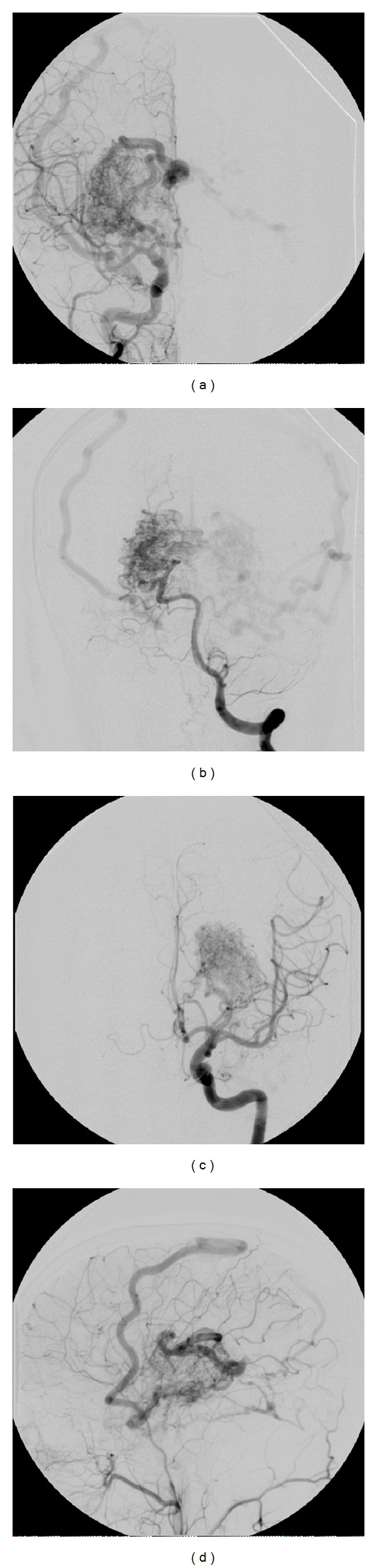
Diagnostic cerebral angiogram at initial presentation. (a) AP right carotid injection. (b) AP left vertebral injection. (c) AP left carotid injection. (d) Lateral right carotid injection.

During his initial hospitalization for further evaluation, an MRI brain (Figure 1) showed evidence of bilateral basal ganglia and thalamic AVMs with evidence of hemorrhage and a small area of encephalomalacia adjacent to the AVM on the right. The digital subtraction angiography (DSA) showed bilateral thalamic and basal ganglia AVMs, Spetzler-Martin grade 4, supplied by the right posterior communicating artery, the P2 and P3 posterior cerebral artery segments, and left anterior choroidal artery. Drainage of the AVM was into the superior sagittal sinus on the right and the superior sagittal sinus, inferior petrosal sinus, and transverse sinus on the left (Figure 2). Presumed seizure activity was empirically treated with levetiracetam despite a negative electroencephalogram (EEG). He was discharged home. He was then evaluated at an outside hospital with plans to observe the AVM.

**Figure 3:**
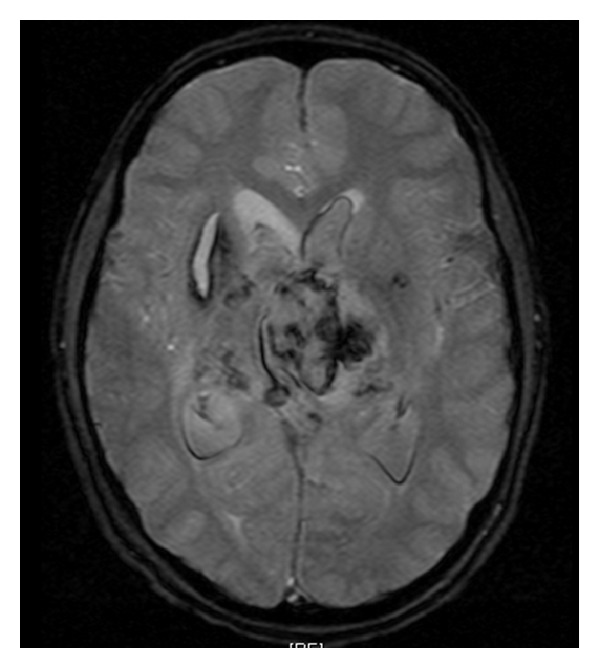
Axial GRE-heme sequence MRI 8 months after diagnosis, during presentation with status epilepticus.

Eight months later, the patient was readmitted for evaluation of seizure activity. A continuous EEG showed no evidence of seizure activity and the levetiracetam was discontinued. Three days after returning home, he presented to the hospital in status epilepticus with a Glasgow Coma Score of 4. After intubation and initiation of antiepileptic medication, an MRI brain showed evidence of intraventricular hemorrhage with hydrocephalus (Figure 3). A ventriculostomy was placed to manage elevated intracranial pressures for approximately 2 weeks until the resolution of the intraventricular hemorrhage. However, the patient developed posthemorrhagic hydrocephalus a week later, necessitating a new ventriculostomy, followed shortly thereafter by a ventriculoperitoneal shunt. The patient was discharged to an inpatient rehabilitation unit after 1 week. He improved to the point that he was awake and alert and could answer some questions with short phrases and follow commands. He expressed emotions of happiness and frustration. He had a diffuse symmetric quadriparesis and could ambulate with a gait belt and assistance. While he could swallow, medications and nutrition were provided primarily by gastrostomy tube. The patient was able to enjoy video games and board games with assistance.

### 2.2. Treatment

**Figure 4:**
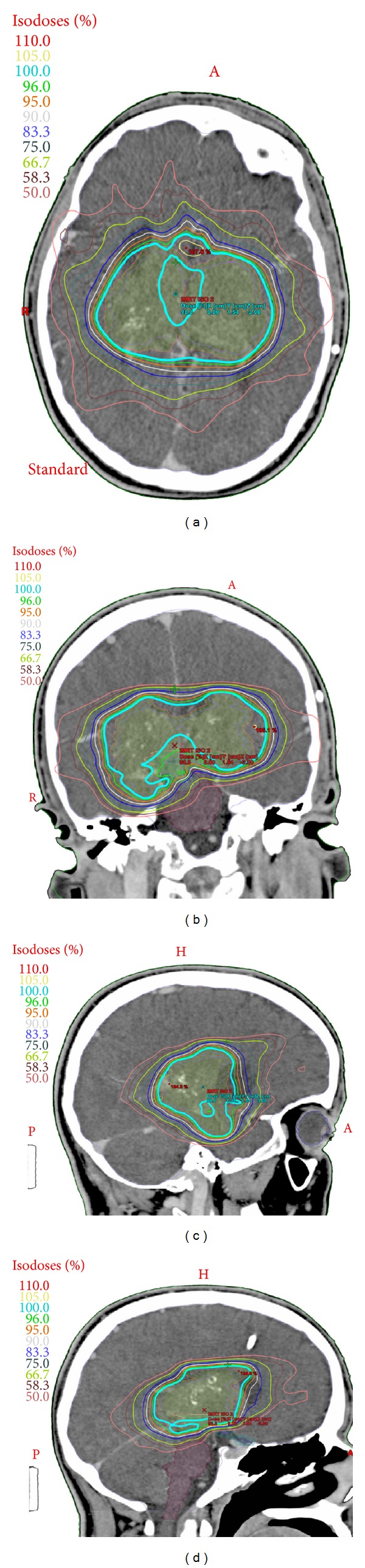
Stereotactic treatment plan. (a) Axial. (b) Coronal. (c) Sagittal left. (d) Sagittal right.

The patient's parents were quite concerned about rehemorrhage and further neurological decline. After much discussion, the family was offered HFSRS to decrease the risk of recurrent hemorrhage. Two months after his discharge to inpatient rehabilitation, the patient underwent frameless stereotactic radiosurgery using the Trilogy Radiosurgery Unit. Pretreatment MRI and CT were obtained for image fusion. Target volumes for each side were drawn separately (left was 34.26 cm^3^ and right was 41.99 cm^3^), as were normal tissue volumes for avoidance. Total prescription volume was 117.64 cm^3^ (Figure 4). A total of 30 Gy was delivered in 5 fractions on an every other day schedule for the first four fractions and 1 week break before the 5th fraction due to hospitalization for seizure. Radiation was delivered with 9 static beams using minimultileaf (MML) collimation with IMRT due to the complex shape of the bilateral target. The patient tolerated the procedure well without complications during treatment.

### 2.3. Postprocedure Course

**Figure 5:**
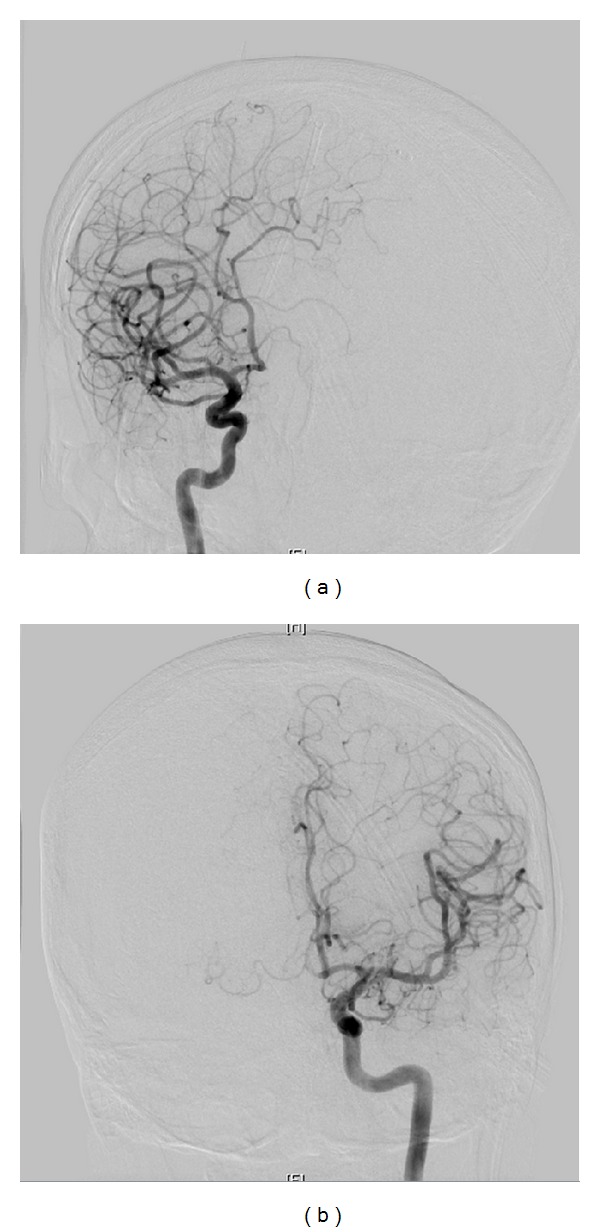
Diagnostic cerebral angiogram 10 months after radiation treatment. (a) AP right carotid injection. (b) AP left carotid injection.

**Figure 6:**
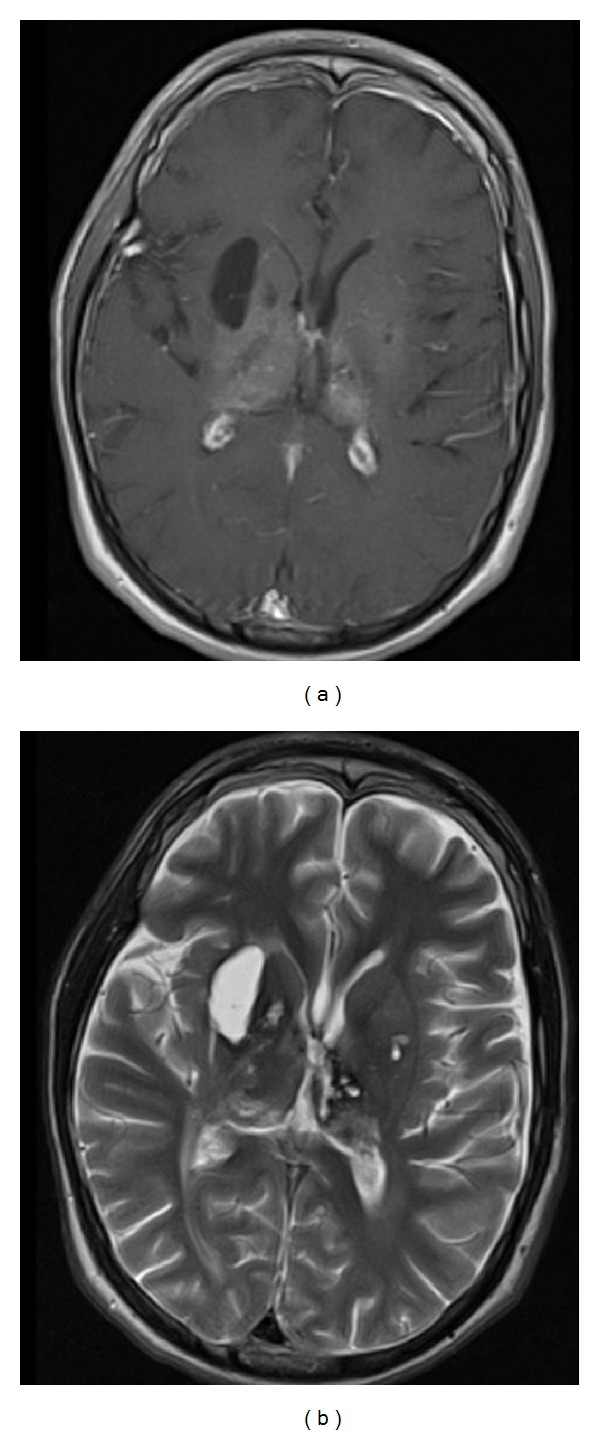
MRI 10 months after radiation treatment. (a) T1-weighted, axial, gadolinium-enhanced image at level of foramen of Monro. (b) T2-weighted, axial image at same level.

The patient was followed closely and had several admissions over the next year for seizure activity. He developed lower extremity contractures requiring Achilles tendon releases. In addition, he developed dystonia and spasticity which was eventually treated with an intrathecal baclofen pump. Repeat DSA (Figure 5) 10 months after radiosurgery showed obliteration of the nidus and absence of filling of the AVMs. The MRI (Figure 6) continued to show cavitary microcystic changes in the area of the prior AVMs. 

Two years after treatment, the patient is able to sit with minimal support, transfer from sitting to standing and back with minimal support, ambulate with a front wheeled walker, and release objects with his hands, and he has normal heel cord range of motion. He has 4+ to 5/5 strength bilaterally and mildly increased tone at the bilateral hip abductors, external rotators, and bilateral ankle plantar flexors. He has continued with home schooling, is able to play simple songs on the piano, and enjoys adaptive gymnastics and swimming, board games, using the Ipad, and playing video games. He still has some dystonic movements; verbal interaction tends to be single words; he nods his head to questions and uses hand gestures to enhance communication. Function varies from day to day, with some good days and others less so; in general, however, the patient has been improving. He has not had any further hemorrhage.

## 3. Discussion

In this report, we describe the successful treatment of a pediatric patient with complete obliteration by HFSRS with IMRT for his large bilateral basal ganglia and thalamic AVM. We chose this technique because no other viable options were really available. The location of the lesion precluded operative intervention, and the large volume precluded single-fraction radiosurgery. Potential ischemic deficits resulting from embolization were a contraindication to this adjunct. While the patient has significant residual weakness and other neurological sequel related to his initial hemorrhage, with potential contributions from subsequent hydrocephalus and seizures, or consequences of his irradiation, he has not had additional hemorrhage since treatment. He has clearly improved from his condition following his hemorrhage, and his parents are pleased that his course has not been complicated by additional episodes of hemorrhage.

The overall incidence and prevalence of intracranial AVMs based on limited population-based studies and autopsy studies are low. Autopsy studies have inherent selection bias, but the reported prevalence of intracranial AVMs is less than 5% [6–9]. Population-based studies have reported an annual incidence of 0.5–2 per 100,000 [6, 10–14]. Deep AVMs in the basal ganglia and thalamus comprise approximately 10% of all brain AVMs [1, 3, 4]. Bilateral deep AVMs are exceedingly rare. Therefore, there are no class I or II studies to determine the best treatment for these lesions. Treatment of AVMs has evolved over the past several decades to include microsurgical, endovascular, and radiosurgical techniques [6]. In general, the decision to treat is based upon the natural history of the disease and the potential benefit of treatment compared with the risk of treatment. 

Based on the known natural history of AVMs, the overall risk of initial hemorrhage of all AVMs ranges between 2% and 5% per year [1, 6, 15–23] with up to a 30% mortality rate from the first hemorrhage [6]. Risk of rehemorrhage during the first year after the initial hemorrhage is reported as high as 25% [24]. Fleetwood et al. [24] found an annual risk of 9.8% in 96 patients with basal ganglia AVMs followed for 55.2 patient-years before surgical management. Sasaki et al. found an 11.4% annual risk of hemorrhage in 15 patients followed for 88 patient-years [25]. In fact, 72–91% of basal ganglia and thalamic AVMs present with hemorrhage [3, 24–27]. These deep AMVs have a more aggressive natural history and present with a higher morbidity [28] and mortality [5] than AVMs in other locations. Hemorrhage-related mortality rates for deep AVMs range from 43% to 71% [5, 25, 29]. Therefore, the goal of treatment is to prevent hemorrhage which may result in death or long-term disability, particularly in these critical locations.

Deep AVMs are considered to have a high morbidity rate with both surgical and endovascular treatments. Morbidity rates following surgical resection in this location range from 13% to 33% [2, 3, 25, 30, 31]. Similarly, embolization of basal ganglia and thalamic AVMs yields a morbidity rate of 11%–40% [3, 25, 32, 33]. Radiosurgery is generally accepted as the first option for treating deep AVMs including the basal ganglia, internal capsule, and thalamus [5, 6, 30, 34–36].

The treatment objective of radiosurgery is complete AVM obliteration in order to reduce the subsequent risk of hemorrhage [3, 5, 6], without causing new neurological deficits. Radiosurgery causes an inflammatory cascade within the blood vessels of the AVM which causes progressive luminal obliteration and thrombosis [37]. This process is generally thought to occur over several years. The risk of hemorrhage is the same to slightly higher after radiosurgery [5, 25] and prior to complete obliteration. Once the AVM is completely obliterated, the hemorrhage risk drops to negligible values. Partial obliteration of the AVM following radiosurgery does not appear to lower the hemorrhage risk [6, 38–40]. Based on case series of deep AVMs, the risk of hemorrhage during the first year after radiosurgery ranges from 0% to 9.5% and decreased to 0%–4.7% in the second year after radiosurgery [3, 5, 25, 29, 41–43]. In one series, multivariate analysis of hemorrhage after radiosurgery showed that a larger target volume was associated with a higher rate of hemorrhage, but margin dose, prior hemorrhage, and prior embolization did not increase the risk of hemorrhage after SRS [3].

Avoidance of adverse radiation effects (AREs)—any new or worsened neurologic symptoms with an onset after radiation not attributed to new hemorrhage [5]—remains a critical factor in determining radiosurgery planning. AREs are considered permanent when lasting for greater than 6 months. Among case series of basal ganglia and thalamic AVMs treated with radiosurgery, AREs were noted in 4%–19% [3, 5, 25, 29, 41–43]. These included worsening of previous motor deficits, quadrantanopsia, hemibody tremor, and partial Parinaud's phenomenon [5]. AVM-associated dystonias have been described in several case reports, but there are no previous reports of radiosurgery-induced dystonia of a basal ganglia or thalamic AVM [44–47]. Yamamoto et al. described one patient who developed a hemi-Parkinson's syndrome 5 years after radiosurgery of a midbrain AVM [48, 49]. Factors associated with a higher risk of symptomatic AREs were thalamic location, larger target volume, larger maximum diameter, lower margin dose, and a higher Pollock-Flickinger score [3].

DSA remains the gold standard to determine obliteration of an AVM. Following radiosurgery, serial MRI is used to assess initial treatment effects. Angiograms are typically obtained no sooner than 3 years after treatment given that the actuarial obliteration rates begin to plateau in that time period [3, 5]. When angiography cannot be obtained, MRI has also been used to assess AVM obliteration; however, MRI can overestimate obliteration rates [3]. The reported obliteration rates of AVMs of the basal ganglia and thalamus treated with single radiosurgery doses of 15–25 Gy ranged from 62% to 86% at 4 to 5 years. The mean volume treated in these series ranged from 1.9 to 3.8 cm^3^ [3, 5, 25, 29, 41–43]. Smaller target volumes, higher marginal dose, and prior hemorrhage are associated with higher obliteration rates [3]. Andrade-Souza et al. [5] defined obliteration of an AVM by the complete disappearance of the nidus, including early filling veins on angiography. The MRI diagnosis of AVM obliteration was defined by the absence of any flow voids in the area of the previously seen AVM, in addition to the lack of visualization of the nidus on MR angiogram.

Prior case series of radiosurgery for treatment of thalamic and basal ganglia AVMs have used LINAC or Gamma Knife in a single fraction [3, 5, 25, 29, 41–43]. Given the large size of the bilateral deep AVM in this patient, single fraction radiosurgery with 20 Gy margin dose would cause unacceptably high treatment-related toxicity. Traditional fractionated daily radiation has generally been ineffective at obliterating AVMs. Lindvall et al. [50] reported a series of 29 patients with a mean AVM volume of 11.5 cm^3^ treated with hypofractionated conformal stereotactic radiotherapy (HCSRT) delivering 30–35 Gy over 5 fractions. This dose staging approach allowed for administering a higher dose of radiation than would have been advisable to administer via single fraction radiosurgery. For AVMs with volumes ranging from 4 to 10 cm^3^ and >10 cm^3^, the obliteration rates were 81% and 70%, respectively, 5 years after treatment. However, four of these patients also received an additional 10–15 Gy 2 years after the initial treatment due to partial obliteration at that time point. In their estimation, a single 18 Gy radiosurgery dose is equivalent to a 32.5 Gy dose delivered in 5 fractions. The authors suggest that AVMs in children may exhibit a higher mitotic and metabolic activity making them particularly sensitive to radiation therapy [50]. Other authors have advocated volume staging [28].

Obviously, although the results obtained in this single patient may not be generalizable to others, HFSRS is an available tool for treating large AVMs in surgically inaccessible locations, with the potential to completely obliterate the lesion. The goal of complete obliteration of the lesion must be carefully weighed against the potential risk of adverse events. Long-term outcome data amongst pediatric patients must continue to be collected as we follow patients into adulthood. Pooled data analyzing the dose-response relationship of radiation treatment of pediatric AVM is needed in order to develop the optimal treatment protocol that is safe and efficient. 

In summary, we successfully treated a large bilateral thalamic and basal ganglia AVM with complete obliteration by HFSRS with IMRT with relatively limited toxicity. Appropriate caution is recommended in applying this technique to other patients.
